# Insights into Triazolylidene Ligands Behaviour at a Di-Iron Site Related to [FeFe]-Hydrogenases

**DOI:** 10.3390/molecules27154700

**Published:** 2022-07-22

**Authors:** Andrea Mele, Federica Arrigoni, Catherine Elleouet, François Y. Pétillon, Philippe Schollhammer, Giuseppe Zampella

**Affiliations:** 1 Laboratoire de Chimie, Electrochimie Moléculaire et Chimie Analytique, UMR 6521 CNRS-Université de Bretagne Occidentale, CS 93837—6 Avenue Le Gorgeu, CEDEX 3, 29238 Brest, France; chemandmele@gmail.com (A.M.); francois.petillon@univ-brest.fr (F.Y.P.); 2 Department of Biotechnology and Bioscience, University of Milano-Bicocca Piazza della Scienza 2, 20126 Milan, Italy

**Keywords:** non-innocent ligand, redox ligand, mesoionic carbene, triazolylidene, DFT calculations, di-iron complexes, metal-sulfur, carbonyle, hydrogenases, bioinspiration

## Abstract

The behaviour of triazolylidene ligands coordinated at a {Fe_2_(CO)_5_(µ-dithiolate)} core related to the active site of [FeFe]-hydrogenases have been considered to determine whether such carbenes may act as redox electron-reservoirs, with innocent or non-innocent properties. A novel complex featuring a mesoionic carbene (MIC) [Fe_2_(CO)_5_(Pmpt)(µ-pdt)] (**1**; Pmpt = 1-phenyl-3-methyl-4-phenyl-1,2,3-triazol-5-ylidene; pdt = propanedithiolate) was synthesized and characterized by IR, ^1^H, ^13^C{^1^H} NMR spectroscopies, elemental analyses, X-ray diffraction, and cyclic voltammetry. Comparison with the spectroscopic characteristics of its analogue [Fe_2_(CO)_5_(Pmbt)(µ-pdt)] (**2**; Pmbt = 1-phenyl-3-methyl-4-butyl-1,2,3-triazol-5-ylidene) showed the effect of the replacement of a n-butyl by a phenyl group in the 1,2,3-triazole heterocycle. A DFT study was performed to rationalize the electronic behaviour of **1**, **2** upon the transfer of two electrons and showed that such carbenes do not behave as redox ligands. With highly perfluorinated carbenes, electronic communication between the di-iron site and the triazole cycle is still limited, suggesting low redox properties of MIC ligands used in this study. Finally, although the catalytic performances of **2** towards proton reduction are weak, the protonation process after a two-electron reduction of **2** was examined by DFT and revealed that the protonation process is favoured by S-protonation but the stabilized diprotonated intermediate featuring a {Fe-H⋯H-S} interaction does not facilitate the release of H_2_ and may explain low efficiency towards HER (Hydrogen Evolution Reaction).

## 1. Introduction

The catalytic site of [FeFe] hydrogenases (H-cluster) contains a di-iron sub-site with an azapropanedithiolate bridge and a cubane [Fe4S4] described as an electron sink, connected together by a sulfur atom of a cysteine [1,2,3]. The high activity of the H-cluster for the electrocatalytic H^+^/H_2_ conversion relies on a fine electronic balance of charges and a well-suited geometry around the di-iron site that are controlled by electron and proton transfers mediated by the amine of the azadithiolate bridge, acting as a proton-relay, and the non-innocent redox [Fe4S4] cluster [4,5,6,7,8].

Carbonyl dithiolato bridged di-iron complexes have been, obviously, considered as the best candidates to mimic the H-cluster in reason of their strong structural resemblance and the high setting possibilities of their coordination sphere in order to modulate their redox and protic properties. For these reasons, such systems have aroused the interest of chemists for several years, allowing novel insights into the organometallic chemistry of di-iron complexes involving bimetallic and metal-ligand cooperativities [9,10,11,12,13,14,15,16,17,18]. In this context, various redox ligands have been combined with such bio-inspired di-iron systems but very few of them display non-innocent behaviour and act as the [Fe4S4] cubane in the H-cluster [19,20,21,22,23,24,25,26,27,28,29,30,31,32,33,34,35]. A few years ago, the intriguing redox non-innocent behaviour of a N-heterocyclic carbene (NHC) was proposed for the two-electron reduction of the complex [Fe_2_(CO)_5_(IMes)(µ-pdt)] (IMes = 1,3-bis(2,4,6-trimethylphenyl)-imidazol-2-ylidene) through a simultaneous dual electron uptake by the di-iron site and the NHC ligand [30].

Among N-Heterocyclic Carbenes ligands, abnormal NHC ligands, also called mesoionic carbenes (MICs), are less reported in the literature [36,37,38,39,40,41,42]. Their canonical zwitterionic nature and the possibility of charge transfer upon bonding to the metal centre raise the question of their possible redox non-innocence and make them interesting for mimicking H-cluster charge transfers.

Recently, we reported the synthesis of the complex [Fe_2_(CO)_5_(Pmbt)(µ-pdt)] (**2**; Pmbt = 1-phenyl-3-methyl-4-butyl-1,2,3-triazol-5-ylidene; pdt = propanedithiolate) in order to investigate the potentially redox non-innocence of such triazolylidene carbenes at a di-iron site [43]. As expected, the ligand Pmbt was found to be a better donor than imidazolin-2-ylidenes. The red-shift of the ν(CO) bands agreed with the evolution of the redox potentials compared to that of the hexacarbonyl complex. Unfortunately, the catalytic activity was non-existent or, at least, very inefficient.

The work presented herein is an extension of our previous results. An analogous complex [Fe_2_(CO)_5_(Pmpt)(µ-pdt)] (**1**; Pmpt = 1-phenyl-3-methyl-4-phenyl-1,2,3-triazol-5-ylidene) has been synthetized in which the aliphatic chain (butyl) of the MIC ligand (in **2**) has been replaced by an aromatic group (phenyl) in order to check the effect of such a modification on the electronic behaviour of the di-iron system. **1** was fully characterized and its electrochemical behaviour was examined by cyclic voltammetry.

DFT calculations were performed for both complexes with the aim to investigate the role of MIC ligands as redox active relay upon one- and two-electron transfers and to rationalize the influence of the modifications of the MIC ligands on the electronic communication between the di-iron core and the carbene. The protonation process after the transfer of two electrons was also considered.

## 2. Results and Discussion

### 2.1. Synthesis and Characterization

The preparation of **1** was realized according to the same protocol used for the synthesis of **2** from the precursor [Fe_2_(CO)_6_(μ-pdt)] in the presence of the Pmpt triazolium salt and a base (potassium tert-butoxide) (see Appendix A) (Figure S1) [43]. The IR spectrum of **1**, recorded in the carbonyl region, in CH_2_Cl_2_, is typical of monosubstituted complexes having a {Fe_2_(CO)_5_L} core, with four bands 2033(s), 1968(vs), 1945(sh), 1910(w) cm^−1^ (Figure S2 in Supplementary Materials). Their comparison with those of **2** (2028(s), 1969(vs), 1950(sh), and 1907(w) cm^−1^ [43]) shows that the replacement in the triazole carbene of the n-butyl chain with a more electron-withdrawing group, such as a phenyl, does not decrease significantly the σ-donor strength of this ligand. This accords with previous observations reported in the literature where the arrangement of the C- vs. N-bound substituents only plays a very slight role for tuning the electronic properties of the metal centre [44,45,46]. Furthermore, calculated ν(CO) values for **1**, **2** (Table S1 in Supplementary Materials) agree with such a trend. **1** has been characterized by ^1^H and ^13^C{^1^H} NMR spectroscopies in CD_2_Cl_2_ (Figures S3 and S4 in Supplementary Materials). These data are in accordance with the molecular structure of **1** described below and close to those of **2**. The ^1^H NMR spectrum of **1** displays in the aromatic region (~7.6–7.7 ppm) the expected signals due to the two phenyls. The N-CH_3_ group is detected at 3.90 ppm as a singlet and CH_2_ of the pdt bridge are typically observed, as multiplets, at 1.78 (2H) and 1.63 (4H) ppm. In the ^13^C{^1^H} NMR spectrum, the two signals at 216.4 (216.6 ppm for **2**) and 212.1 ppm (211.8 ppm for **2**) are attributed to the carbonyls of the {Fe(CO)_3_-Fe(CO)_2_} core. The peak at 173.3 ppm (171.2 ppm for **2**) is typical of the carbenic carbon of the triazolylidene ligand linked to one iron atom. Other signals (see Appendix A) are assigned to the second carbon of the 1,2,3-triazole cycle, propanedithiolate, and N-Me groups.

**Figure 1:**
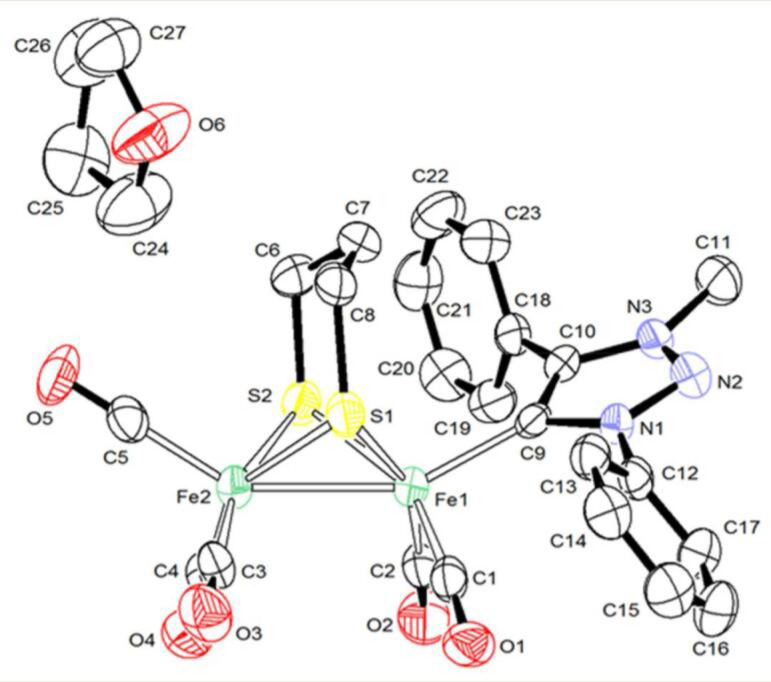
Ortep view (ellipsoids at 30% of probability level) of **1.C_4_H_8_O**. Selected distances (Å) and angles (deg): C9-Fe1, 1.980(5); Fe1-Fe2, 2.5275(11); S1-Fe1, 2.2781(16); S1-Fe2, 2.2726(15); S2-Fe2, 2.2704(17); S2-Fe1, 2.2707(15); C9-N1, 1.389(5); C9-C10, 1.409(7); C11-N3, 1.466(6); N1-N2, 1.343(5); N2-N3, 1.320(5); C10-N3, 1.366(6); C12-N1, 1.430(6); C1-O1, 1.166(6); C1-Fe1, 1.744(6); C2-O2, 1.135(6); C2-Fe1, 1.778(6); C3-O3, 1.137(6); C3-Fe2, 1.787(6); C4-O4, 1.158(6); C4-Fe2, 1.774(6); C5-O5,1.154(6); C5-Fe2, 1.777(6); Fe1-S1-Fe2, 67.48(5); Fe2-S2-Fe1, 67.64(5); C5-Fe2-Fe1, 146.56(18); C9-Fe1-Fe2, 151.97(14); C1-Fe1-Fe2, 100.29(18); C2-Fe1-Fe2, 103.24(17); C3-Fe2-Fe1, 107.34(18); C4-Fe2-Fe1, 95.30(18); O1-C1-Fe1, 175.2(5); O2-C2-Fe1, 178.1(5); O3-C3-Fe2, 178.6(5); O4-C4-Fe2, 178.7(5); and O5-C5-Fe, 177.4(5).

Complex **1** was crystallized, as red single crystals, from slow evaporation of a solution hexane/THF (1:1) at −30 °C and its structure was determined by X-ray diffraction analysis (Figure 1 and Table S2 in Supplementary Materials). The Pmpt ligand lies in apical position, in contrast to Pmbt in **2**, which is in basal position, but similarly to the structure observed for other analogous imidazole-derived carbene complexes [30,31,33]. The apical position of the Pmpt ligand, that is observed experimentally for **1,** accords with the more stable forms determined by DFT calculations (apical isomer is more stable by 1.7 kcal/mol than the basal form). For **2**, the basal disposition of Pmbt has been confirmed as the most stable isomer, since the apical isomer is 2.7 kcal/mol higher in energy. This may reflect the different steric effects of the n-butyl and phenyl groups.

The Fe-C(_MIC_) distance (1.980(5) Å) is very close to that observed in **2** (1.9839(19) Å) and those reported for other NHC-monosubstituted di-iron complexes [Fe_2_(CO)_5_L(µ-pdt)] [33]. The Fe-Fe length (2.5275(11) Å) is in the range of distances expected for single Fe(I)-Fe(I) bond in complexes [Fe_2_(CO)_6-x_L_x_(µ-pdt)] [9]. A typical eclipsed geometry is observed around the two iron atoms which have a square pyramidal coordination geometry. The overall geometry of **1** is unexceptional and other X-ray data (distances and angles) will not be further described.

The electrochemical behaviour of **1** in CH_2_Cl_2_-[Bu_4_N][PF_6_] 0.2 M is comparable to that of **2**. Indeed, **1** undergoes an irreversible reduction according to an ECE mechanism and a chemically reversible oxidation. The potential values are close to those of **2**: *E*_p_^c, red^ = −2.28 V vs. (Fc^+^/Fc) (−2.29 V for **2**) and *E*_1/2_^ox^ = −0.02 V (0.02 V for **2**) at 0.2 V s^−1^ (Figure S5 in Supplementary Materials) indicating, as suggested previously by the IR analysis, that the replacement of the n-butyl group by the electron-withdrawing phenyl does not modify significantly the redox behaviour of **1**–**2**. The theoretical redox potential of the first electron transfer is −2.25 V for **1** and −2.28 V for **2**, in good agreement with the experimental values. The redox potential computed for the second electron transfer is −2.17 V and −2.16 V for **1** and **2**, respectively, indicating that this electron transfer is thermodynamically easier than the first, which also matches the electrochemical observations showing a two-electron reduction at slow scan rates (Figure S5c in Supplementary Materials).

### 2.2. DFT Investigations of the Geometries of the Mono-Anion and Di-Anion

In order to shed some light on the possible redox behaviour of the triazolylidene ligand in **1**–**2**, the stereoelectronic features of the mono-anion and di-anion were investigated (Table S3 in Supplementary Materials).

The isomeric speciation of elusive mono-anion (**1**^−^ and **2**^−^) and of di-anion (**1**^**2**−^ and **2**^**2**−^) has been carried out to identify the structures of these ions that are likely present in solution. The flipping central methylene of propanedithiolate bridge is not predicted to significantly alter the thermodynamic speciation of reduced isomers (both **1**^−^ and **2**^−^), so it has been neglected (however, we checked that the methylene flip has an associated ΔG = 0.8 kcal/mol and 0.2 kcal/mol for the neutral **1** and **2**, respectively).

**Figure 2:**
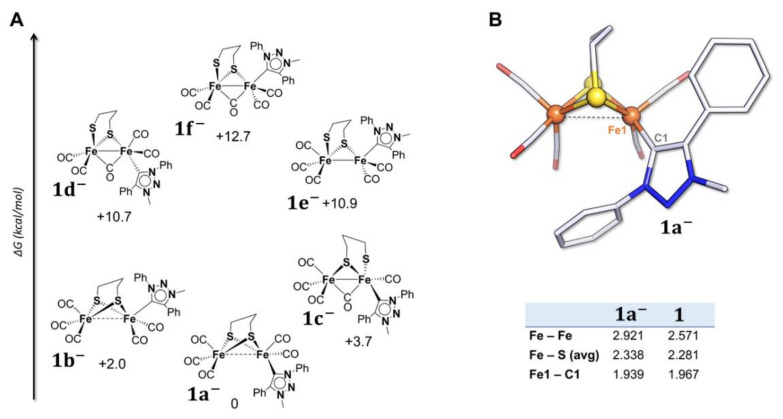
(**A**) Thermodynamic speciation of **1**^−^ and (**B**) optimized structure of **1a**^−^ with relevant geometrical parameters (distances in Å) in comparison with the ones of **1**. Atom coloring: Fe = orange, S = yellow, C = grey, N = blue, and O = red. H atoms are omitted for clarity. For simplification, net charges on structures are not reported.

The relative stabilities of different geometries of **1**^−^ (and **2**^−^) are reported in Figure 2A (Figure S6 in Supplementary Materials, for **2**^−^).

**Figure 3:**
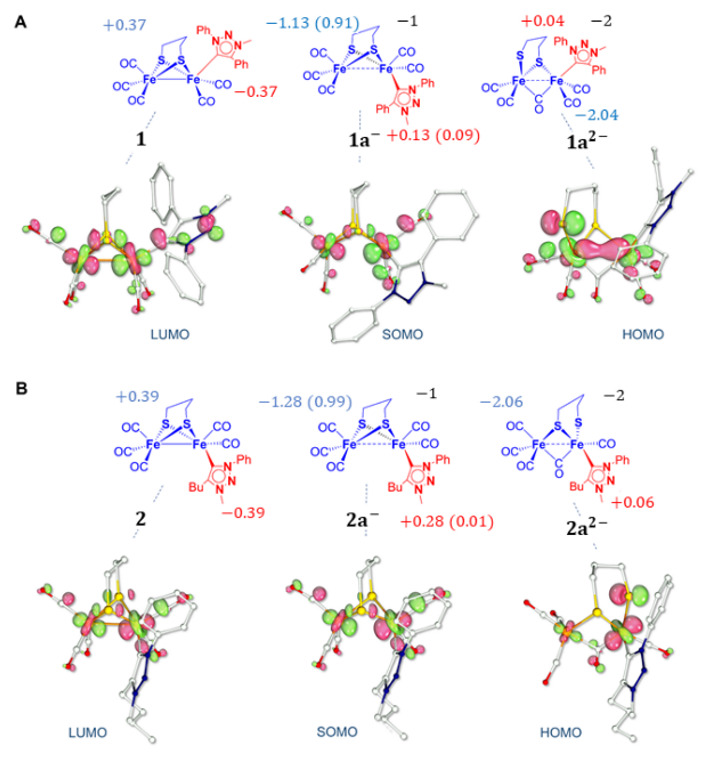
Natural Population Analysis (NPA) charges on the Pmpt (**A**) and Pmbt (**B**) ligands (red values) and on the rest of the complex for most stable neutral, anionic, and dianionic forms of **1** (**A**) and **2** (**B**). Spin densities (in electrons) calculated for **1a**^−^ and **2a**^−^ are reported in parenthesis. Bottom: Selected FMOs for the three redox states (isosurface cutoff: 0.05 a.u.).

The most stable computed structure of **1**^−^ closely resembles that of **2**^−^, where the only structural variation observed is the elongation of the Fe-Fe bond. Other structural changes, such as Fe-S bond breaking or bridging of CO, are energetically prohibited or do not correspond to a minimum on the potential energy surface. Upon one-electron transfer of **1**, the triazolylidene preferentially adopts a basal position (the isomer **1a**^−^ is 2 kcal/mol lower in energy than **1b**^−^, the isomer with the Pmpt ligand in the apical position). In the case of **2**^−^, there are no notable changes in the geometry of the complex and the Pmbt ligand keeps its basal position. The main difference between **1**^−^ and **2**^−^ is a slightly more elongated Fe-Fe distance in **1**^−^: a lengthening of 0.347 Å and 0.332 Å is observed in **1**^−^ and **2**^−^, respectively. This is due to the antibonding character of the SOMO (Figure 3) of both mono-anions.

The analysis of the SOMO of **1**^−^ and **2**^−^ shows that the molecular orbitals have a pure Fe_2_S_2_ character suggesting that the electron density is not located on the triazolylidene ligands upon one-electron transfer. Spin density calculation predicts that the unpaired electron is entirely delocalized on the bimetallic core, without participation of the Pmpt or Pmbt ligands, with a higher density on the iron atom bonded to the carbene and a strong orbital contribution of the two sulfur atoms. This is consistent with the LUMO shape and the electronic distribution in the neutral complexes, which is confined to the Fe-S region.

The DFT IR spectrum of **1**^−^ (Table S1 in Supplementary Materials) also shows strict analogy with that of **2**^−^. The average value of the ν(CO) is 1889 cm^−1^ (1870 cm^−1^ for **2**^−^) and agrees with the expected red-shift compared to the neutral complex. The extent of the shift, 76 cm^−1^, is a bit less than that observed for **2**^−^ (94 cm^−1^), suggesting that the donor strength of the Pmpt ligand, in **1**^−^, is slightly weaker than that of Pmbt, in **2**^−^. Moreover, the extent of this shift is consistent with a strong enhancement of the electron density within the di-iron unit of the complexes which is confirmed by the spin density analysis (Figure 3). Indeed, the stereoelectronic features of both mono-anions suggest that MIC ligands do not play an active role in hosting electron density upon the first reduction since the extra electron density almost completely populates the di-iron core.

**Figure 4:**
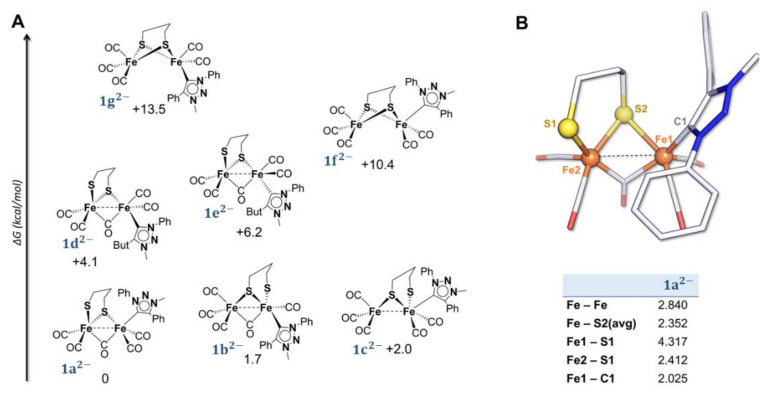
(**A**) thermodynamic speciation of **1^2^**^−^ and (**B**) optimized structure of **1a^2^**^−^ with relevant geometrical parameters (distances in Å). Atom coloring: Fe = orange, S = yellow, C = grey, N = blue, and O = red. H atoms are omitted for clarity. For simplification, net charges on structures are not reported.

The different geometries considered for the structure of **1^2^**^−^ are reported in Figure 4. Upon the second electron transfer, the breaking of one Fe-S bond is observed in the most stable isomer, **1a^2^**^−^. The resulting coordination vacancy is compensated by the migration of one basal CO to a bridging position between the two metal centres. This overall rearrangement is consistent with the electrochemical behaviour of **1**. The two distorted squared pyramidal geometries are held together by the bridging CO bond and one Fe-S bond, even if a weak iron–iron interaction is still present.

The calculated structure of **1^2^**^−^ (Figure 4) displays expected similarities to that of **2^2^**^−^ (Figure S7 in Supplementary Materials). Nevertheless, it has to be noted that the cleaved Fe-S bond in **1^2^**^−^ is different from that in **2^2^**^−^. Indeed, the breaking of one Fe-S bond can lead to different isomers since the two iron atoms are differently substituted. In the case of **1^2^**^−^, the most stable geometry presents a Fe-S bond cleavage with the {Fe(CO)_2_(Pmpt)} moiety, which is different from what is observed for **2^2^**^−^ (Figure S7 in Supplementary Materials). Regardless of their different stereochemistry, the Fe-S cleavage in the two doubly reduced complexes is consistent with the ECE mechanism proposed on the CV basis. The theoretical IR spectrum of **1^2^**^−^ (Table S1 in Supplementary Materials) is similar to that of **2^2^**^−^ with a weak band at 1657 cm^−1^ (1646 cm^−1^ for **2^2^**^−^) that can be assigned to the asymmetrical stretching of the µ-CO. A red-shift of 88 cm^−1^ (91 cm^−1^ for **2^2^**^−^) for the average value of ν(CO) (1801 cm^−1^) (1780 cm^−1^ for **2**^2−^) compared to that of **1**^−^ (**2**^−^) is expected. Such a strong shift suggests that the second electron transfer is centred at the Fe_2_S_2_ core. It is noteworthy that the HOMO of **1^2^**^−^ (Figure 3A) is different from that calculated for **2^2^**^−^. It has mainly a Fe_2_S_2_ character with no contribution of the Pmpt ligand and it can be described as a combination of a σ_FeFe_ bonding orbital with a σ*_FeS_ contribution. The HOMO of **2^2^**^−^, reported in Figure 3B, has also an almost pure Fe_2_S_2_ character with no involvement of the Pmbt ligand, but it corresponds to an overall σ*_2Fe2S_ orbital with two different d_Fe_ orbitals and a σ*_Fe-S_ contribution. The charge distribution within the complex is −2.04 for **1^2^**^−^ (−2.06 for **2^2^**^−^) on the iron core and +0.04 on the Pmpt ligand (+0.06 on Pmbt). Comparing the negative charge distribution of the di-anion to that computed for the mono-anion and neutral form, one can conclude that the di-iron core hosts most of the electron density upon the first and second electron transfers. The triplet spin state (S = 1), with an unpaired electron at the Fe-S region and one distributed over Pmpt, is 14 kcal/mol (13.6 kcal/mol for Pmbt) less stable than the singlet spin state (S = 0), further stressing the low tendency of Pmpt (Pmbt) to accommodate electron density upon reduction.

### 2.3. In Silico Modification of the MIC Ligand

**Figure 5:**
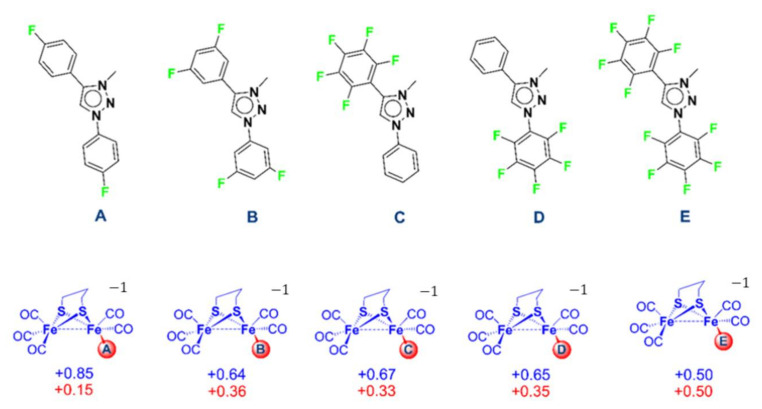
Structure of the Pmpt ligand variants tested (**A**–**E**, top), with the corresponding spin density values (in electrons, bottom) calculated for the anionic form of the corresponding mono-substituted complexes (spin density on the modified Pmpt ligands is reported in red, while the one on the rest of the complex comprising the Fe_2_S_2_ core is in blue).

The Pmpt carbene, even with two electron-withdrawing phenyl groups, turns out to not be likely to host additional electron density from reduction. Indeed, the electron transfers are exclusively Fe_2_S_2_ centred for both the neutral and anionic forms due to the absence of hybrid metal ligand molecular orbital establishing electronic communication. Further modifications of the MIC ligand have been considered in a silico DFT approach to predict how the relevant stereoelectronic features of such di-iron species could be modulated. The DFT structure of **1**^−^ has been modified by systematically adding an increasing number of powerful electron-withdrawing element/group such as fluorine atoms in different positions on the aromatic rings of the Pmpt ligand (Figure 5, top). The spin density values have been calculated (Figure 5, bottom) to determine the distribution of the unpaired electron density over the entire molecular structure.

The data show that the systematic replacement of hydrogen by fluorine atoms in the phenyl rings of the triazolylidene should trigger and then proportionally increase the delocalization of the electron density in the reduced (anionic) form. A significant electron delocalization is only obtained with, at least, four fluorine atoms (**B**, **C**, and **D** forms). Moreover, it has to be emphasized that even with a fully perfluorinated Pmpt ligand, the electron transfer is not exclusively located on the triazolylidene, suggesting that a genuine non-innocence of such carbene ligands is questionable and still an open question.

### 2.4. DFT Investigations of Protonation and Electron Steps in the Proposed Electrocatalytic Pathway

Despite the fact that the electrocatalytic activities of **2** towards proton reduction are inefficient or non-existent, DFT calculations were performed, using **2** as a model of di-iron site linked to a MIC ligand, to find out which electron and proton transfers are energetically preferred in the processes involving such species. Indeed, since di-iron complexes featuring MIC or NHC ligands have comparable activity and redox behaviour, they may similarly be involved in the electrocatalytic HER process [30,33,47,48]. This approach may highlight the reactivity flaws of such species and inspire the design of more efficient complexes with MIC or NHC ligands. The study of the catalytic cycle for the proton reduction was carried out (DFT methodological details are provided in Materials and Methods).

**Figure 6:**
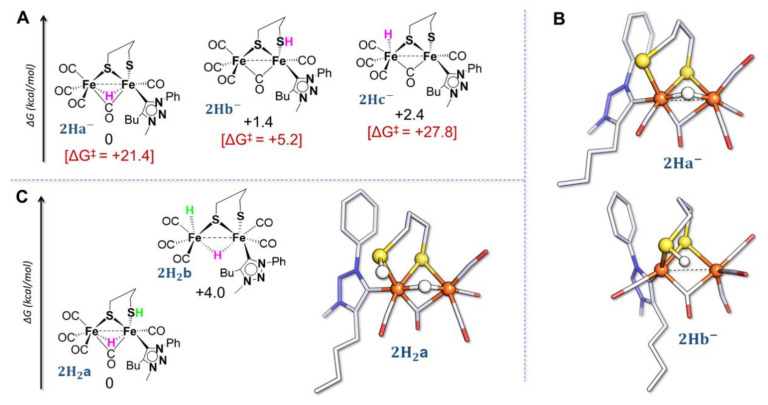
(**A**) thermodynamic speciation of **2H**^−^. Furthermore, the activation barrier associated with the formation of each protonated form are reported (red values). (**B**) optimized structure of thermodynamic (**2Ha**^−^) and kinetic (**2Hb**^−^) products of the first protonation. (**C**) thermodynamic speciation of **2H_2_** and optimized structure of **2Ha_2_**. Atom coloring: Fe = orange, S = yellow, C = grey, N = blue, and O = red. H atoms are omitted for clarity. For simplification, net charges on structures are not reported.

First, three geometries have been considered for the species **2H**^−^ arising from the protonation of **2^2^**^−^ (Figure 6A).

The breaking of one Fe–S bond leads to a possible S-protonation in reason of the available lone pair (Figure 6A,B, **2Hb**^−^). The barriers of activation for the processes leading to the three geometries have been calculated and it turns out that the direct formation of hydride species (**2Ha**^−^—in which the hydride is coordinated to the two metal centres in a bridging mode and **2Hc**^−^—in which the hydride is terminally coordinated to a single iron atom) by protonation of the di-iron site is kinetically rather impeded. The mutual comparison of energy barriers (Figure 6A) suggests that the first step of the mechanism is probably the S-protonation (lowest activation barrier), followed by an intramolecular proton transfer to the di-iron site, generating the bridging hydride species, thermodynamically more stable. The value of the energy barrier for this proton migration {Fe_2_SH} → {Fe_2_(µ-H)} (**2Hb**^−^ → **2Ha**^−^) is +14.2 kcal/mol. It may involve the formation of the transient terminal hydride species **2Hc**^−^ that finally isomerizes into the bridging hydride form **2Ha**^−^. The computed structure of the thermodynamic product **2Ha**^−^ is mainly characterized by the presence of a bridging hydride in a quasi-symmetrical coordination between the two iron atoms (Fe1–H = 1.694 Å, and Fe2–H = 1.665 Å). The geometry around both iron atoms is octahedral, with an evident distortion of the dithiolate bridge. In the S-protonated species **2Hb**^−^, the S-H bond distance is 1.367 Å while it is 1.495 Å in the transition state. The ΔG estimated for the protonation is −10.8 kcal/mol with a ΔG^‡^ of +5.2 kcal/mol, consistently with a facile and fast reaction.

Two possible structures have been found for the species arising from the second protonation (Figure 6C). The DFT structure of the thermodynamically stable neutral di-protonated species **2H_2_a** resembles the previous structure proposed for **2Ha**^−^ but with a protonated S atom. The structure of the second isomer, **2H_2_b**, is characterized by the presence of a bridging hydride and a terminal hydride at the {Fe(CO)_3_} moiety and the absence of a bridging CO. **2H_2_a** is notably more stable than the dihydride form. The S–H length is 1.353 Å and the distance between the two H atoms is 2.773 Å. If the species **2H_2_a** is considered in the proton reduction process, the final formation of H_2_ would involve a proton–hydride coupling {S-H⋯H-Fe}. However, the H⋯H distance and the unfavorable spatial orientation require a strong distortion in the metal coordination sphere for the formation and the release of H_2_. The value of the energy barrier is indeed +18.1 kcal mol^−1^ which is rather high (the computed rate constant is 4.0 s^−1^). This result agrees with the essentially low (or even absent) catalytic activity of such a complex.

**Figure 7:**
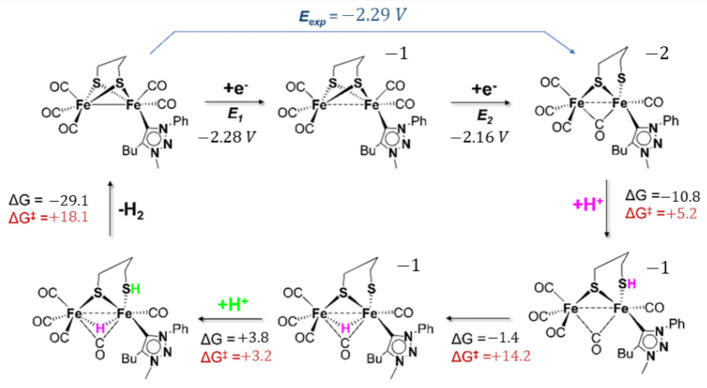
Proposed catalytic cycle for proton reduction.

Figure 7 resumes the putative DFT mechanism of proton reduction mediated by **2**.

The two electron transfers centred on the di-iron core trigger the cleavage of a Fe-S bond with the concomitant bridging of a CO between iron atoms. DFT calculations suggest that the first protonation of the mechanism is a S-protonation, leading to the isomer **2Hb**^−^ that, subsequently, isomerizes to the thermodynamically more stable isomer **2Ha**^−^ with a bridging hydride.

A further S-protonation affords **2H_2_a** that is able through a proton–hydride interaction {S-H⋯H-Fe} to release H_2_. This last step is predicted to be the rate-determining step due to the relatively high value of the energy barrier (vide supra). Due to the lack of electronic communication between the ligand and the di-iron core in **2**^−^ and **2^2^**^−^ and the absence of any obvious involvement during the catalytic cycle, it can be proposed that the ligand Pmbt, in **2**, has a redox innocent behaviour.

Finally, the poor catalytic activity (if any) towards proton reduction of the Pmbt derivative may be associated with a slow formation and release of H_2_, probably due to a regiochemically impeded H^+^-H^−^ hetero-coupling during the last step of the catalytic cycle.

## 3. Materials and Methods

All the experiments were carried out under an inert atmosphere, using Schlenk techniques for the syntheses. Solvents were deoxygenated and dried according to standard procedures. The iron precursor [Fe_2_(CO)_6_(µ-pdt)] [49] and Pmpt = 1-phenyl-3-methyl-4-phenyl-1,2,3-triazol-5-ylidene [42] were prepared according to literature procedures. All other reagents were commercially available and used as purchased. NMR spectra (^1^H and ^13^C) were recorded with Bruker DRX500 spectrometer of the “Service général des plateformes, Université de Bretagne Occidentale, Brest” and were referenced to SiMe_4_ (^1^H) and H_3_PO_4_ (^31^P). The infrared spectra were recorded on a FT IR VERTEX 70 Bruker spectrometer. Chemical analyses were made by the “Service de Microanalyse I.C.S.N.”, Gif sur Yvette (France). Electrochemical measurements were conducted using a PG-STAT 128 N Autolab or a µ-autolab (type III) electrochemical analyzer driven by the GPES software. All the electrochemical studies were carried out in a conventional three-electrode cell under an inert atmosphere (argon). The preparation and the purification of the supporting electrolyte [Bu_4_N][PF_6_] were as described previously [50]. The working electrode was a vitreous carbon disk of 0.3 cm in diameter, polished with alumina prior to use. A platinum wire was used as counter electrode. The reference electrode was an Ag|Ag^+^ electrode, however, all the potentials (text, tables, and figures) are quoted against the (Fc^+^/Fc) couple; ferrocene was added as an internal standard at the end of the experiments. Crystal data for compound **1** were collected on an Oxford Diffraction X-Calibur-2 CCD diffractometer, equipped with a jet cooler device and graphite-monochromated Mo-Kα radiation (λ = 0.71073 Å). The structure was solved and refined by standard procedures [51]. Deposition number CCDC 2179366 contain the supplementary crystallographic data for **1**. These data can also be obtained free of charge from the Cambridge Crystallographic Data Centre via www.ccdc.cam.ac.uk/data_request/cif (accessed on 15 June 2022).

Calculations have been performed using the TURBOMOLE 7.4.1 suite of programs [52]. A triple-ζ TZVP basis (for all atoms) and the pure functional BP86 were used [53,54,55]. This particular basis-set/functional combination has been widely validated in the context of Fe/Ni-thiolate bioinorganic and biomimetic systems, also in relation to hydrogenases [56,57,58,59]. The Resolution-of-Identity (RI) technique allowed us to speed up geometry optimizations [60]. Grimme’s corrections with Becke–Johnson damping (D3BJ) were added to account for dispersive interactions [61,62]. COSMO was used to implicitly treat the solvent, by setting a ε_r_ = 8.93 of CH_2_Cl_2_ [63,64]. The nature of each stationary point was verified by means of full vibrational analysis. Atomic charges and spin densities were calculated performing a Natural Population Analysis (NPA) as implemented in the Natural Bond Orbital (NBO) procedure. The total partition function (Q) has been evaluated as a product of q-rotational, q-translational, and q-vibrational contributions and used to derive free energies from SCF energy values, by setting temperature and pressure to 298.15 K and 1 bar, respectively (the scaling factor for the SCF wavenumbers has been set to 0.9914, as default value in TURBOMOLE for the adopted theoretical scheme). In-solvent free energies were used to calculate absolute redox potentials of the couple under investigation, according to the relation E° = −ΔG°/nF (*n* = number of electrons involved in the redox process; F = Faraday constant), which were then scaled by a reference value (−5.08 V for the Fc^+^/Fc couple, calculated at the same level of theory).

## 4. Conclusions

The two complexes [Fe_2_(CO)_5_(MIC)(µ-pdt)] (**1**,**2**) have been prepared and studied in order to describe the effect of mesoionic carbenes on the behaviour of such di-iron systems. The two triazolylidenes, which were used, differ by a phenyl group (in **1**) instead of a butyl group (in **2**). This change affects the relative stability of basal, apical isomers related to the position of the carbene. The different DFT-predicted stable isomers, with apical and basal position of this ligand, are observed in solid state for **1** and **2**, respectively. Spectroscopic and electrochemical characterization show the close and good σ-donation ability of these triazole-based carbenes, by increasing the electron density of the di-iron site. DFT investigations addressed the question of the redox, innocent or non-innocent, properties of these ligands by examining the electronic structure of mono- and di-anion of **1**,**2**. They show that MIC ligands lying in the first coordination sphere of a carbonyl di-iron dithiolate core feature innocent redox behaviour. The replacement of the butyl by a phenyl group in the triazole cycle is insufficient to trigger the electronic communication between the triazolylidene and the di-iron core. Moreover, only significant perfluorination of the phenyl groups carried by the triazole cycle allows the partial distribution of the electronic density over various atomic centres. This observation may afford interesting perspectives for the design of new triazole systems endowed with a non-innocent redox behaviour.

Finally, the importance of the sulfur environment is highlighted by DFT when considering the protonation processes after the transfer of two electrons. On the one hand, S-protonation facilitates the formation of required hydride species for HER evolution, affording a proton relay but, on the other hand, intermediates featuring {Fe⋯H⋯H⋯S} do not favor the release of H_2_, suggesting the reason why **1**,**2** are not efficient for HER catalysis.

## Data Availability

The data presented in this study are available in supplementary material.

## Supplementary Materials

The following supporting information can be downloaded at: https://www.mdpi.com/article/10.3390/molecules27154700/s1, Figure S1: Geometry of complexes **1**, **2**; Figure S2: IR spectrum of **1** in CH_2_Cl_2_ (ν(CO) region); Figure S3: ^1^H NMR spectrum of **1** in CD_2_Cl_2_; Figure S4: ^13^C{^1^H} NMR spectrum of **1** in CD_2_Cl_2_; Figure S5: (a) Cyclic voltammograms of **1** (1.9 mM), CH_2_Cl_2_-[NBu_4_][PF_6_] (0.2 M) under Ar at 0.2 V s^−1^. (b) Variation of the current intensity (*I*p) as a function of the square root of the scan rate (ν^1/2^). (c) Scan rate dependence of the current for the reduction of 1 in CH_2_Cl_2_-[NBu_4_][PF_6_] (0.2 M) (vitreous carbon electrode); Figure S6: (A) thermodynamic speciation of **2**^−^ and (B) optimized structure of **2a**^−^ with relevant geometrical parameters (distances in Å) in comparison with the ones of its neutral counterpart **2**. Atom coloring: Fe = orange, S = yellow, C = grey, N = blue, and O = red. H atoms are omitted for clarity. For simplification, net charges on structures are not reported.; Figure S7: (A) thermodynamic speciation of **2**^**2**−^ and (B) optimized structure of **2a**^**2**−^ with relevant geometrical parameters (distances in Å). Atoms coloring: Fe = orange, S = yellow, C = grey, N = blue, and O = red. H atoms are omitted for clarity. For simplification, net charges on structures are not reported; Table S1: Calculated ν(CO) (cm^−1^) of complexes **1** and **2** and their most stable mono-anion and di-anion of **1** and **2**; Table S2: Crystallographic data and refinement parameters of **1.C_4_H_8_O**; Table S3: XYZ Coordinates of selected optimized structures.

## Sample Availability

Samples of the compounds are not available from the authors.

## Appendix A

*Synthesis of [Fe_2_(CO)_5_(Pmpt)(µ-pdt)] (**1**)*

A solution of [Fe_2_(CO)_6_(µ-pdt)] (200 mg, 0.518 mmol) with an excess of 1-phenyl-3-methyl-4-phenyl-1,2,3-triazol-5-ylidene, Pmpt, (300 mg, 1.28 mmol) was prepared in degassed anhydrous THF (25 mL). 144 mg of potassium tert-butoxide (1.28 mmol) were added at 0 °C under vigorous stirring and the mixture was stirred for 30 min. The solvent was evaporated under reduced pressure to dryness and then 10 mL of CH_2_Cl_2_ were added. The resulting red solution was filtered over Celite and evaporated to dryness to yield **1** as a red powder (184 mg, 60%). Crystallization by slow diffusion at −35 °C (hexane/THF 1:1) afforded red single crystals suitable for X-ray diffraction analysis.

IR (CH_2_Cl_2_, cm^−1^): ν(CO) 2033(s), 1968(vs), 1945(sh), 1910(w)

^1^H NMR (CD_2_Cl_2_, δ, ppm): δ 7.70; 7.60; 7.57 (m, 10H, N-Ph and Ph), 3.90 (s, 3H, CH_3_), 1.78 (2H, m, pdt), 1.63 (4H, m, pdt)

^13^C-{^1^H} NMR (CD_2_Cl_2_, δ, ppm): 216.4 (s, CO), 212.1 (s, CO), 173.3 (s, Fe-CMIC), {149.6, 140.9, 131.8, 130.4, 129.1, 128.4} (C_6_H_5_ + N-C_6_H_5_), 37.60, 29.9, 24.3 (NCH_3_ + S(CH_2_)_3_S)

Elemental Analysis calculated for C_23_H_19_Fe_2_N_3_O_5_S_2_: 46.54% C, 3.20% H, 7.08% N. Found: 46.11% C, 3.33% H, 6.98% N.
