# Supplementary material for: Insights into Triazolylidene Ligands Behaviour at a Di-Iron Site Related to [FeFe]-Hydrogenases
Source: Molecules. 2022 Jul 22;27(15):4700. doi: 10.3390/molecules27154700 (PMC9369626; doi:10.3390/molecules27154700)
Supplement: Supplementary file 1 [file molecules-27-04700-s001.zip › molecules-1820938-supplementary.pdf]

Supplementary materials

Figure S1. Geometry of complexes **1, 2**.

Figure S2. IR spectrum of **1** in CH_2Cl_2 (ν(CO) region)

Figure S3. ^1H NMR spectrum of **1** in CD_2Cl_2

Figure S4. $^{13}C\{^1H\}$ NMR spectrum of **1** in CD_2Cl_2

Figure S5. (**a**) Cyclic voltammograms of **1** (1.9 mM) in CH_2Cl_2-$[Bu_4N][PF_6]$ (0.2 M) under Ar at 0.2 Vs^{-1}. (**b**) Variation of the current intensity (*I*p) as a function of the square root of the scan rate ($v^{1/2}$). (**c**) Scan rate dependence of the current for the reduction of **1** in CH_2Cl_2-$[Bu_4N][PF_6]$ (0.2M) (vitreous carbon electrode).

Figure S6. **A**) thermodynamic speciation of $\mathbf{2}^-$ and **B**) optimized structure of $\mathbf{2a}^-$ with relevant geometrical parameters (distances in Å) in comparison with the ones of its neutral counterpart **2**. Atom colouring: Fe = orange, S = yellow, C = grey, N = blue, O = red. H atoms are omitted for clarity. For simplification, net charges on structures are not reported.

Figure S7. A) thermodynamic speciation of $\mathbf{2}^{2-}$ and **B**) optimized structure of $\mathbf{2a}^{2-}$ with relevant geometrical parameters (distances in Å). Atoms colouring: Fe = orange, S = yellow, C = grey, N = blue, O = red. H atoms are omitted for clarity. For simplification, net charges on structures are not reported.

Table S1. Calculated ν(CO) (cm^{-1}) of complexes **1, 2** and their most stable mono-anion and di-anion

Table S2. Crystallographic data and refinement parameters of **1.C_4H_8O.**

Table S3. XYZ Coordinates of selected optimized structures.

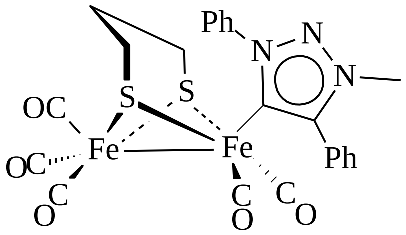

1

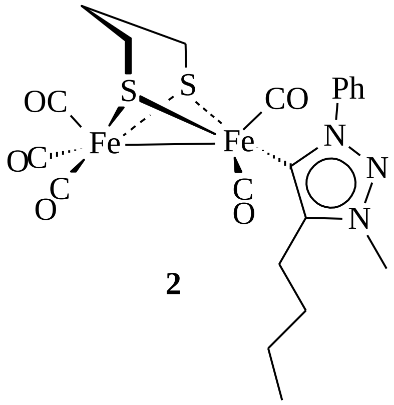

2

Figure S1. Geometry of complexes **1, 2**.

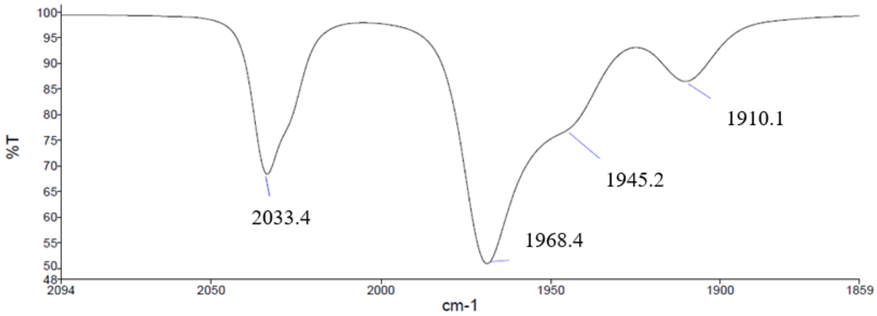

Figure S2. IR spectrum of **1** in CH_2Cl_2 (ν(CO) region)

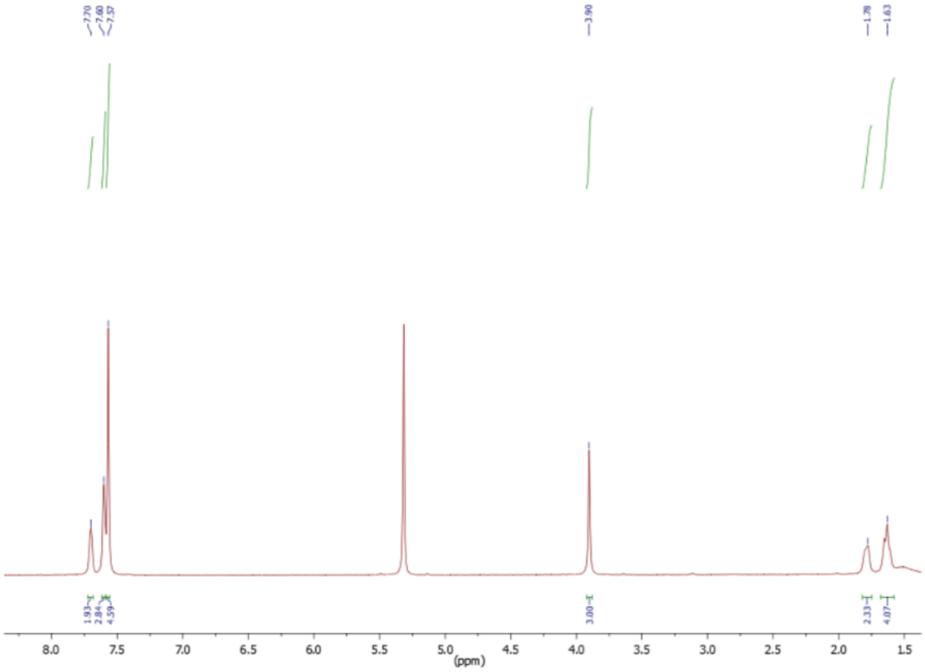

Figure S3. ^{1}H NMR spectrum of **1** in CD_2Cl_2

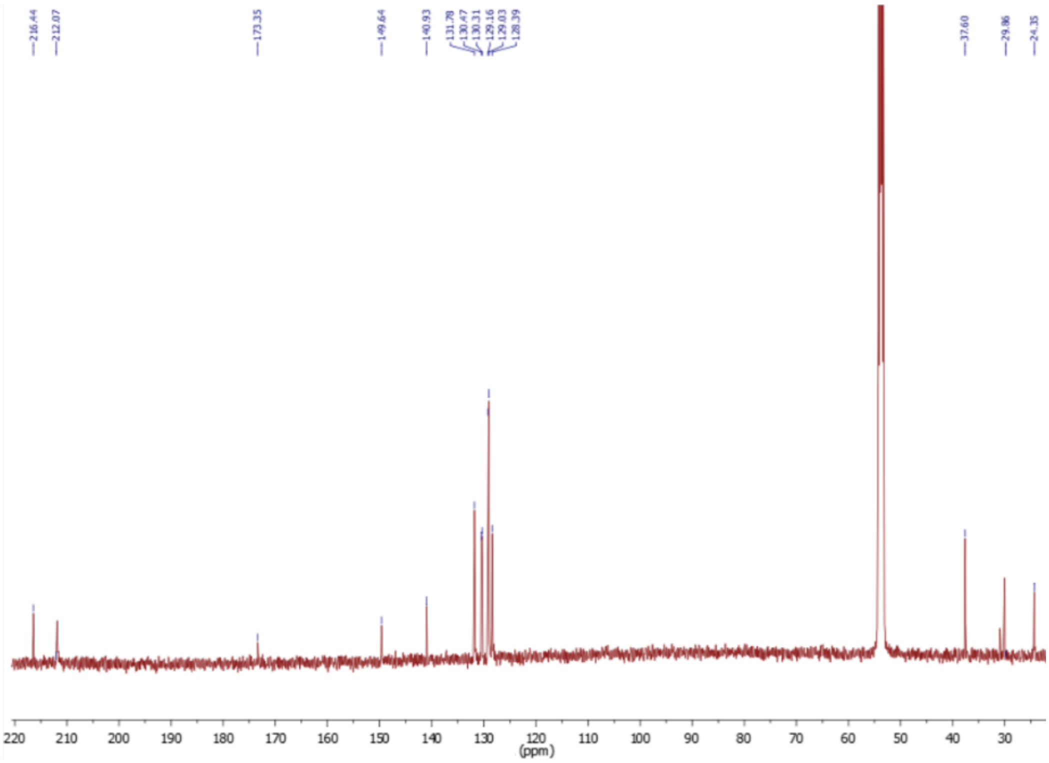

Figure S4. $^{13}C\{^1H\}$ NMR spectrum of **1** in CD_2Cl_2

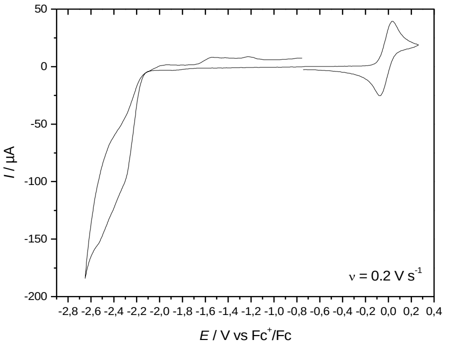

(a)

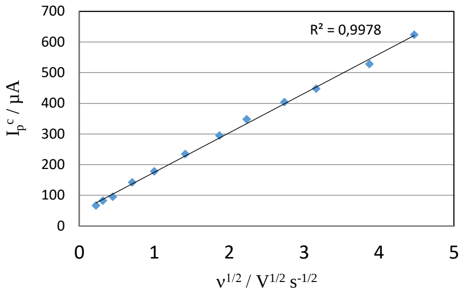

(b)

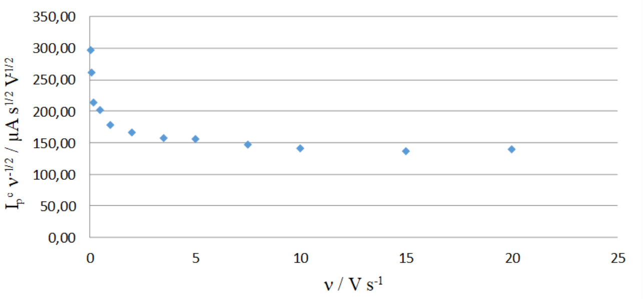

(c)

Figure S5. (**a**) Cyclic voltammograms of **1** (1.9 mM) in CH_2Cl_2-[Bu_4N][PF_6] (0.2 M) under Ar at 0.2 Vs^{-1}. (**b**) Variation of the current intensity (*I*p) as a function of the square root of the scan rate ($\nu^{1/2}$). (**c**) Scan rate dependence of the current for the reduction of **1** in CH_2Cl_2-[Bu_4N][PF_6] (0.2M) (vitreous carbon electrode).

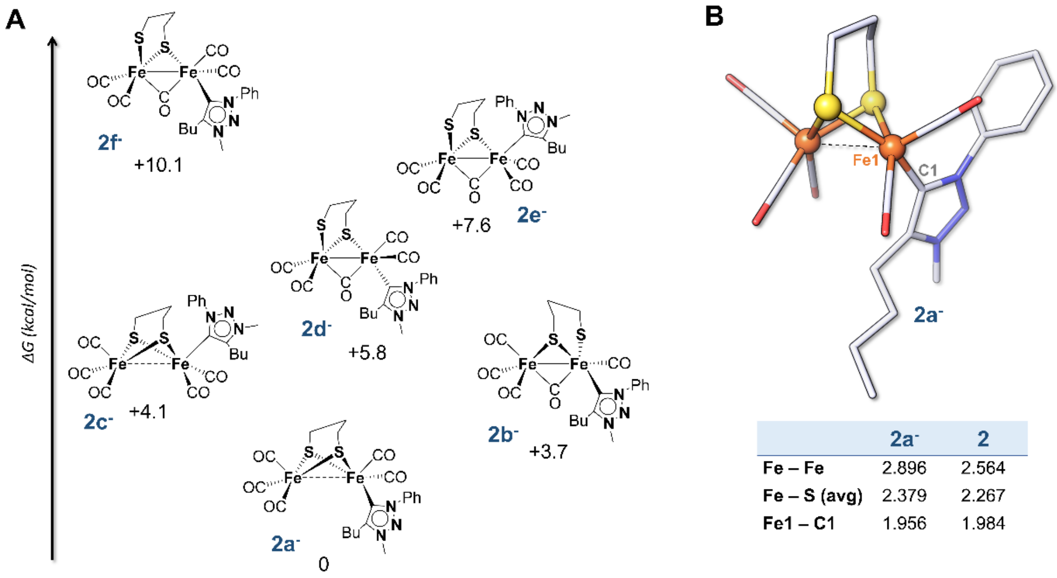

	$2a^-$	2
Fe – Fe	2.896	2.564
Fe – S (avg)	2.379	2.267
Fe1 – C1	1.956	1.984

Figure S6. **A**) thermodynamic speciation of **2**$^-$ and **B**) optimized structure of **2a**$^-$ with relevant geometrical parameters (distances in Å) in comparison with the ones of its neutral counterpart **2**. Atom colouring: Fe = orange, S = yellow, C = grey, N = blue, O = red. H atoms are omitted for clarity. For simplification, net charges on structures are not reported.

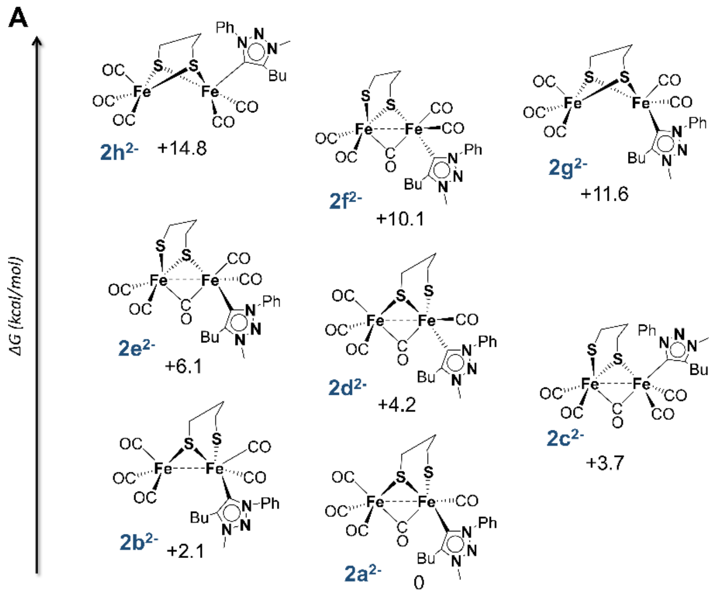

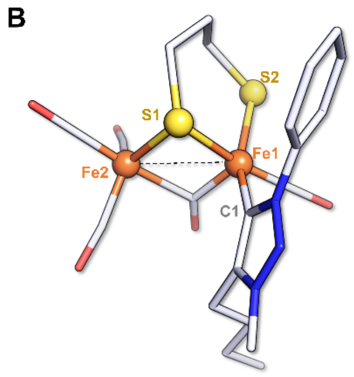

	$2a^{2-}$
**Fe – Fe**	2.918
**Fe – S1(avg)**	2.371
**Fe1 – S2**	2.379
**Fe2 – S2**	3.994
**Fe1 – C1**	1.945

Figure S7. **A**) thermodynamic speciation of $\mathbf{2}^{2-}$ and **B**) optimized structure of $\mathbf{2a}^{2-}$ with relevant geometrical parameters (distances in Å). Atoms colouring: Fe = orange, S = yellow, C = grey, N = blue, O = red. H atoms are omitted for clarity. For simplification, net charges on structures are not reported.

Table S1. Calculated ν(CO) (cm^{-1}) of complexes **1**, **2** and their most stable mono-anion and di-anion.

**species**	ν(CO) / cm^{-1}
**1**	1920, 1944, 1958, 1963, 2016
$\mathbf{1^{-}}$	1852, 1854, 1884, 1894, 1933
$\mathbf{1^{2-}}$	1657, 1791, 1825, 1844, 1886
**2**	1914, 1944, 1947, 1962, 2010
$\mathbf{2^{-}}$	1818, 1857, 1860, 1877, 1927
$\mathbf{2^{2-}}$	1646, 1783, 1811, 1821, 1869

Table S2. Crystallographic data and refinement parameters of $\mathbf{1.C_4H_8O}$

		$\mathbf{1.C_4H_8O}$
Empirical formula		$C_{27}H_{27}Fe_2N_3O_6S_2$
Formula weight		665.34
Temperature (°K)		170(2)
Wavelength (Å)		0.71073
Crystal system, space group		Triclinic, P-1
Unit cell dimensions	a (Å)	9.1679(8)
	b (Å)	9.7446(9)
	c (Å)	16.3731(14)
	α (°)	99.169(7)
	β (°)	97.263(2)
	γ(°)	94.410(7)
Volume ($Å^3$)		1424.9(2) $Å^3$
Z		2
Calculated density (Mg/m^3)		1.551
Absorption coefficient (mm^{-1})		1.211
F(000)		684
Crystal colour		Red
Crystal size (mm)		0.18 x 0.08 x 0.01
Theta range for data collection (°)		3.25 to 26.37
Limiting indices		$-9 \leq h \leq 11$, $-12 \leq k \leq 10$, $-17 \leq l \leq 20$
Reflections collected / unique		9784 / 5817 [R(int) = 0.0847]
Completeness to theta = 26.37 (%)		99.7
Absorption correction		Analytical
Max. and min. transmission		0.9880 and 0.8115
Refinement method		Full-matrix least-squares on F^2
Data / restraints / parameters		5817 / 0 / 362
Goodness-of-fit on F^2		0.938
Final R indices [I>2σ(I)]		$R_1 = 0.0636$, $wR_2 = 0.0956$
R indices (all data)		$R_1 = 0.1275$, $wR_2 = 0.1242$
Largest diff. peak and hole (e.$Å^{-3}$)		0.679 and -0.587

Table S3. XYZ Coordinates of selected optimized structures.

2

C	4.938346	5.239871	4.259887
C	3.207701	4.094470	2.723169
C	1.370404	6.066821	0.929079
C	0.400623	7.251738	2.974927
C	1.899799	8.770558	1.282333
C	5.268087	7.984426	1.810301
H	6.201326	7.628939	1.353661
H	4.880957	8.810929	1.198017
C	5.512219	8.421556	3.243295
H	5.933221	7.586760	3.820819
H	6.278547	9.215876	3.232648
C	4.280600	8.959592	3.947580
H	3.863470	9.830993	3.424407
H	4.527991	9.260547	4.975211
C	2.125477	5.109689	4.943036
C	0.852978	4.515227	4.852164
C	0.131653	3.943896	3.683223
H	0.608843	4.356639	2.786201
H	-0.910815	4.301082	3.671429
C	0.153678	2.405981	3.616509
H	1.193088	2.055329	3.709872
H	-0.399042	1.983010	4.471082
C	-0.448691	1.889283	2.307381
H	0.110009	2.327276	1.463871
H	-1.485238	2.255882	2.215267
C	-0.427571	0.362582	2.206066
H	0.602468	-0.022130	2.258537
H	-0.866209	0.017865	1.258359
H	-0.998508	-0.097247	3.027471
C	-0.989149	4.029740	6.540978
H	-1.215138	4.471321	7.515391
H	-0.962229	2.936848	6.620566
H	-1.744714	4.332136	5.807958
C	3.290762	6.023893	7.009288
C	3.176603	7.363717	7.384434
H	2.275461	7.920983	7.134182
C	4.239280	7.968506	8.059028
H	4.166778	9.016946	8.350173
C	5.392211	7.232311	8.356597
H	6.222937	7.709754	8.877964
C	5.478747	5.884325	7.994707
H	6.372150	5.307394	8.234544
C	4.420779	5.270455	7.319181
H	4.471689	4.222651	7.026138
N	0.316211	4.528333	6.112442
N	1.123400	5.062937	7.019797
N	2.207732	5.399432	6.296501
O	6.012182	4.940664	4.616700
O	3.124950	3.060337	2.186181
O	0.969779	5.317588	0.132313
O	-0.621309	7.283506	3.531697
O	1.855760	9.780065	0.703165
S	4.086010	6.570372	1.578766
S	2.885095	7.758006	4.159062
Fe	3.398318	5.636499	3.535756
Fe	1.960573	7.218012	2.140070

$2a^-$

C	5.110047	5.211432	4.319062
C	3.322962	3.957354	2.798160
C	1.262310	6.149850	0.875663
C	0.314028	7.282220	2.893092
C	1.905220	8.874942	1.277227
C	5.174094	7.919247	1.828173
H	6.130806	7.592620	1.395754
H	4.770508	8.733592	1.208114
C	5.383079	8.380339	3.262593
H	5.787920	7.547544	3.855681
H	6.147538	9.178484	3.259214
C	4.135771	8.912939	3.950001
H	3.703205	9.754120	3.388372
H	4.387373	9.257061	4.964389
C	2.221523	5.003814	4.999358
C	0.910616	4.501101	4.870551
C	0.215998	3.947719	3.678505
H	0.746778	4.349771	2.805096
H	-0.814491	4.334943	3.626547
C	0.200722	2.410010	3.618199
H	1.232603	2.040524	3.721881
H	-0.370619	1.999896	4.468592
C	-0.394335	1.899720	2.303418
H	0.181857	2.329732	1.467633
H	-1.424314	2.281367	2.197340
C	-0.393843	0.372359	2.202373
H	0.630279	-0.025097	2.274659
H	-0.819585	0.031689	1.246966
H	-0.985303	-0.080095	3.013876
C	-1.049576	4.244423	6.466428
H	-1.266893	4.688687	7.441672
H	-1.138132	3.152923	6.521936
H	-1.745139	4.634409	5.714912
C	3.332197	5.976575	7.051769
C	3.197279	7.304333	7.461110
H	2.280383	7.845332	7.233064
C	4.261191	7.919726	8.125979
H	4.171086	8.960002	8.441759
C	5.441142	7.207259	8.372339
H	6.273625	7.693697	8.883470
C	5.554410	5.872828	7.967862
H	6.471869	5.315748	8.160744
C	4.494399	5.249748	7.303325
H	4.566251	4.215178	6.971747
N	0.311785	4.616356	6.095317
N	1.108802	5.135102	7.026118
N	2.254016	5.350626	6.343254
O	6.199085	4.846889	4.610080
O	3.297221	2.903060	2.263494
O	0.857353	5.395747	0.068266
O	-0.720413	7.291051	3.454152
O	1.734347	9.876463	0.677119
S	4.013256	6.481911	1.598289
S	2.778274	7.673160	4.184025
Fe	3.513577	5.484401	3.612213
Fe	1.856140	7.289338	2.068453

$2a^{2-}$

C	2.723291	4.031805	3.867553
C	1.194198	5.489242	2.288219
C	-0.945464	7.149238	2.169794
C	1.589820	7.423327	0.618220
C	0.537025	9.303940	2.028731
C	4.626238	7.777383	2.397046
H	5.607603	7.862403	1.906322
H	3.854119	8.122213	1.692718
C	4.592292	8.629140	3.670459
H	5.275067	9.493234	3.559719
H	4.965920	8.026447	4.513068
C	3.214493	9.180857	4.006095
H	2.884018	9.886055	3.230413
H	3.256255	9.722832	4.963376
C	1.021702	5.415323	5.188387
C	-0.279327	4.880851	5.270518
C	-1.133785	4.293479	4.205497
H	-1.008799	4.910940	3.308459
H	-2.195966	4.351244	4.490958
C	-0.754003	2.842635	3.863204
H	0.311544	2.823689	3.593457
H	-0.877191	2.199172	4.752471
C	-1.579230	2.295204	2.697835
H	-1.441836	2.968780	1.836776
H	-2.652154	2.325553	2.956955
C	-1.178429	0.869798	2.309498
H	-0.118355	0.831009	2.015498
H	-1.776027	0.496092	1.464362
H	-1.315805	0.173854	3.152497
C	-1.945902	4.536090	7.156459
H	-1.882060	4.672802	8.239574
H	-2.755527	5.153891	6.749463
H	-2.131178	3.481400	6.922563
C	2.417427	6.394571	7.065600
C	2.262574	7.367988	8.057082
H	1.263841	7.628833	8.404990
C	3.393659	8.019109	8.558294
H	3.275154	8.789406	9.322073
C	4.666302	7.699974	8.071136
H	5.545384	8.221747	8.453361
C	4.805902	6.714507	7.086974
H	5.792546	6.458953	6.698096
C	3.684919	6.050965	6.586353
H	3.777132	5.313864	5.788256
N	-0.671421	4.950615	6.578978
N	0.247777	5.481089	7.382014
N	1.261830	5.751408	6.524350
O	3.028199	2.885738	3.998781
O	0.818164	4.598284	1.556191
O	2.048313	7.337118	-0.476249
O	-2.110312	6.951886	1.995588
O	0.401251	10.478195	1.932808
S	4.367555	5.977152	2.700021
S	1.854204	7.930461	4.231384
Fe	2.286472	5.676717	3.734524
Fe	0.749440	7.573310	2.136631

1

C	3.805549	8.072808	3.204037
C	4.172401	6.874940	5.327847
C	0.718958	8.801030	3.632486
C	1.120550	7.841379	5.951840
C	-0.824008	6.677690	4.345491
C	1.146765	3.783254	4.205717
H	1.538520	2.921365	4.761206
H	0.058134	3.822938	4.351576
C	1.507654	3.676497	2.736023
H	2.599557	3.630863	2.626748
H	1.105778	2.724165	2.349771
C	0.967795	4.805229	1.879469
H	-0.126533	4.878884	1.949043
H	1.233369	4.646836	0.825374
C	4.585652	5.283549	3.133523
C	5.006048	3.997197	3.540130
C	6.794006	2.374294	2.699032
H	6.269630	1.586563	2.146274
H	6.937937	2.067622	3.739505
H	7.757638	2.585470	2.226755
C	5.459749	6.664080	1.189572
C	4.429349	6.884828	0.275903
H	3.594164	6.189175	0.221115
C	4.483193	8.015080	-0.540917
H	3.679314	8.204797	-1.252757
C	5.564506	8.898408	-0.446144
H	5.600996	9.782535	-1.083785
C	6.598434	8.652348	0.463642
H	7.440191	9.341455	0.537730
C	6.550574	7.527680	1.290726
H	7.336508	7.325822	2.018407
C	4.520332	3.103963	4.599905
C	4.502946	3.518241	5.941301
H	4.893403	4.498591	6.206625
C	4.000438	2.673144	6.932232
H	3.986894	3.008764	7.969780
C	3.511468	1.406294	6.596894
H	3.111347	0.751440	7.372215
C	3.537747	0.980170	5.264197
H	3.153657	-0.004376	4.994165
C	4.036833	1.824086	4.270923
H	4.024039	1.499920	3.229579
N	5.409099	5.506297	2.041148
N	6.274838	4.513837	1.755797
N	6.007729	3.608834	2.682483
O	4.085819	9.155769	2.869522
O	4.774460	7.128688	6.297807
O	0.604714	9.865516	3.177713
O	1.284051	8.282264	7.016250
O	-1.943379	6.355378	4.374055
S	1.624458	6.495864	2.269808
S	1.845517	5.247879	5.102062
Fe	3.275765	6.535699	3.867759
Fe	0.877019	7.177289	4.327069

$1a^{-}$

Fe	3.582549	6.914352	4.316609
Fe	0.824167	7.177838	3.393161
S	1.735353	5.713954	4.955596
C	1.762491	4.036024	4.153074
C	2.722531	3.869303	2.983554
C	2.464965	4.775461	1.790704
S	2.715763	6.591768	2.123503
C	3.549815	7.790701	5.827434
O	3.613217	8.261765	6.909487
C	4.779146	8.173178	3.453613
C	6.015628	7.943109	2.810652
N	6.451055	9.143340	2.313595
N	5.617044	10.143206	2.566895
N	4.620515	9.535883	3.255048
C	6.759871	6.692380	2.610635
C	8.053791	6.520343	3.125707
C	8.726463	5.308347	2.951340
C	8.113273	4.257292	2.262014
C	6.826432	4.427044	1.738441
C	6.150887	5.633643	1.917228
C	3.500529	10.367229	3.609952
C	2.583057	10.701056	2.614042
C	1.514026	11.542212	2.930982
C	1.374855	12.038782	4.230472
C	2.305470	11.697017	5.217538
C	3.380167	10.859988	4.909186
C	7.672606	9.416538	1.558588
C	4.793849	5.708696	4.750748
O	5.535089	4.966904	5.299511
C	0.424058	8.284432	2.102853
O	0.137278	8.969725	1.192095
C	0.581579	8.343383	4.694711
O	0.314292	9.077283	5.573854
C	-0.565421	6.134430	3.132557
O	-1.578447	5.532066	3.045242
H	2.027503	3.330431	4.953628
H	0.725558	3.833230	3.847839
H	3.750320	4.039250	3.335573
H	2.665778	2.818803	2.644660
H	1.438204	4.660246	1.414524
H	3.161827	4.519982	0.978581
H	7.860781	8.569397	0.890749
H	8.521106	9.542091	2.241567
H	7.516582	10.332844	0.981801
H	2.701524	10.291522	1.611123
H	0.783070	11.799606	2.163966
H	0.533117	12.687898	4.476469
H	2.188742	12.073952	6.234647
H	4.112019	10.588560	5.667598
H	8.525126	7.324409	3.691680
H	6.343690	3.610731	1.198862
H	5.141210	5.776253	1.533769
H	9.724662	5.181052	3.372631
H	8.634369	3.306389	2.138384

$1a^{2-}$

N	5.777761	3.535560	2.588987
C	5.006487	3.966504	3.644626
C	4.647625	5.322058	3.400718

N	5.245214	5.512595	2.151306
N	5.942955	4.461934	1.656017
Fe	3.769325	6.746265	4.541326
Fe	1.146517	7.478401	3.735652
S	1.726362	5.576739	4.929933
C	1.474537	3.965724	4.053732
C	1.630360	3.937015	2.541040
C	0.474557	4.599101	1.789770
S	0.596273	6.435568	1.631155
C	4.640248	3.054237	4.731455
C	4.684804	3.479826	6.069529
C	4.277079	2.628834	7.097665
C	3.827443	1.334764	6.810460
C	3.787452	0.896759	5.482032
C	4.183525	1.750409	4.450922
C	5.205435	6.683390	1.326360
C	3.970656	7.276828	1.059435
C	3.941022	8.419263	0.256792
C	5.124097	8.956353	-0.264486
C	6.351885	8.341466	0.006821
C	6.397154	7.198592	0.812801
C	6.472896	2.262318	2.401635
C	4.664101	8.086652	3.892055
O	5.284613	9.051519	3.593143
C	4.483494	6.719318	6.118030
O	4.991275	6.877353	7.187072
C	1.320949	8.956073	2.835867
O	1.414316	9.983935	2.259457
C	-0.338276	7.845327	4.531629
O	-1.325508	8.183480	5.101573
C	2.407726	8.187674	5.011184
O	2.445438	9.153925	5.744886
H	2.183036	3.277553	4.528795
H	0.459293	3.638542	4.329952
H	2.579044	4.414944	2.256826
H	1.695770	2.874965	2.236226
H	-0.475884	4.328964	2.280733
H	0.432149	4.211569	0.760001
H	5.842408	1.558402	1.845223
H	6.713988	1.845233	3.383917
H	7.390442	2.461240	1.839166
H	3.042523	6.861245	1.464395
H	2.979881	8.892047	0.050819
H	5.090399	9.855013	-0.883630
H	7.277361	8.756439	-0.395860
H	7.345037	6.717115	1.054210
H	5.019840	4.490832	6.284742
H	4.311131	2.979690	8.130537
H	3.503650	0.673388	7.615950
H	3.423584	-0.104275	5.244121
H	4.090701	1.417898	3.416777
